# Trachoma Prevalence and Associated WASH and Behavioral Risk Among Rural Communities of North Gondar Zone, Ethiopia: A Community‐Based Cross‐Sectional Study

**DOI:** 10.1155/jotm/8924826

**Published:** 2026-07-29

**Authors:** Gebre Ayanaw Alula

**Affiliations:** ^1^ Department of Biology, College of Natural and Computational Science, Debark University, Debark, Ethiopia, dku.edu.et

**Keywords:** active trachoma, North Gondar Zone, rural Ethiopia, SAFE strategy, trachoma, WASH

## Abstract

Trachoma is a leading infectious cause of preventable blindness and remains a major public health problem in rural Ethiopia, where inadequate water, sanitation, and hygiene (WASH) conditions sustain transmission. This study assessed the prevalence of active trachoma and associated WASH and hygiene‐related factors among children aged 1–9 years in rural communities of North Gondar Zone, Ethiopia. A community‐based cross‐sectional study was conducted among 456 children selected using a multistage sampling technique. Data on sociodemographic characteristics, household WASH conditions, and hygiene practices were collected using structured caregiver interviews and direct observation. Active trachoma was diagnosed using the WHO simplified trachoma grading system (trachomatous inflammation–follicular and/or trachomatous inflammation–intense). Poor face‐washing practice was defined as washing the child’s face less than twice per day or the presence of ocular or nasal discharge, while good practice was defined as washing the child’s face at least twice per day and the absence of ocular or nasal discharge. High fly density was defined as the observation of three or more eye‐seeking flies on or around the child’s face during household assessment. Binary and multivariable logistic regression analyses were performed, with statistical significance set at *p* < 0.05. The overall prevalence of active trachoma was 20.6% (95% CI: 16.9%–24.3%). In multivariable analysis, higher odds of active trachoma were observed among children from households using unimproved water sources (AOR = 1.76; 95% CI: 1.01–3.05), households without latrines (AOR = 1.94; 95% CI: 1.15–3.28), children with poor face‐washing practices (*A* *O* *R* = 2.63; 95% CI: 1.52–4.55), and children exposed to high fly density (AOR = 2.71; 95% CI: 1.60–4.58). These findings indicate significant associations rather than causal relationships due to the cross‐sectional design. The study highlights the continued burden of trachoma in the area and supports strengthening integrated WASH and facial cleanliness interventions under the WHO SAFE strategy.

## 1. Introduction

Trachoma is the leading infectious cause of preventable blindness worldwide and remains a major public health concern, particularly in low‐income settings [1]. It is responsible for an estimated 1.9 million cases of visual impairment, including approximately 1.4 million people with irreversible blindness [2]. The disease is caused by repeated ocular infection with *Chlamydia trachomatis*, resulting in chronic conjunctival inflammation, scarring, trichiasis, and eventual blindness if untreated [3]. Although the World Health Organization (WHO) has promoted the SAFE strategy (surgery, antibiotics, facial cleanliness, and environmental improvement), transmission continues in communities where inadequate water, sanitation, and hygiene (WASH) conditions persist [4, 5].

Ethiopia remains one of the most trachoma‐endemic countries globally and is a priority for elimination efforts [6, 7]. The disease is particularly concentrated in rural settings, where limited access to improved water sources, inadequate sanitation infrastructure, and overcrowded living conditions create favorable environments for transmission [8]. North Gondar Zone is among the known endemic areas; however, recent community‐level evidence on both disease burden and associated risk factors remains limited [9, 10].

Although several studies in Ethiopia have examined trachoma prevalence and associated factors, important methodological gaps remain [9, 11–13]. In particular, WASH conditions have been measured inconsistently across studies, and hygiene‐related indicators such as facial cleanliness, frequency of face washing, soap use, and caregiver‐reported hygiene practices have not been uniformly defined or standardized. Furthermore, few studies have integrated the WHO/United Nations Children’s Fund (UNICEF) Joint Monitoring Program (JMP) standardized WASH classification framework with systematically measured household hygiene indicators within a single analytical model in highly endemic rural settings such as North Gondar Zone [14, 15].

This limitation restricts a comprehensive understanding of how structural WASH conditions and household hygiene practices jointly contribute to trachoma transmission and may weaken the evidence base for targeted intervention strategies [16]. Addressing this gap is essential for strengthening programmatic planning for trachoma elimination.

Therefore, this study aimed to determine the prevalence of active trachoma and identify associated WASH conditions and hygiene‐related practices among rural communities of North Gondar Zone, Ethiopia, using a community‐based cross‐sectional design. The study applied standardized WHO/UNICEF JMP WASH classifications alongside clearly operationalized hygiene indicators to generate more comparable and policy‐relevant evidence for trachoma control strategies.

## 2. Methods

### 2.1. Study Area and Period

This study was conducted in North Gondar Zone, Amhara Regional State, Northwest Ethiopia, located approximately 830 km from Addis Ababa and about 103 km from Gondar town. The zone lies between 13°8′ N latitude and 37°54′ E longitude and is characterized by diverse topography, including highland and lowland areas, with elevations reaching about 2850 m above sea level.

North Gondar Zone has a predominantly rural population of over 900,000 people, whose livelihoods mainly depend on subsistence agriculture. The study area experiences climatic variation with temperatures ranging approximately from 12°C to 20°C. The study was conducted from January to April 2025.

### 2.2. Study Design

A community‐based cross‐sectional study design was employed to assess the prevalence of active trachoma and its associated WASH conditions, and hygiene‐related risk factors among rural households in North Gondar Zone, Ethiopia. This design allows for the simultaneous measurement of the outcome (active trachoma) and exposure variables at a single point in time, enabling the assessment of associations between disease status and potential risk factors at the population level.

### 2.3. Source and Study Population

The source population comprised all rural households in North Gondar Zone. The study population consisted of households selected from randomly chosen rural kebeles within the zone.

Within each selected household, one child aged 1–9 years was included for clinical eye examination, as this age group is the standard indicator population for active trachoma surveillance. In households with more than one eligible child, one child was selected using a simple random sampling technique.

Caregivers who were permanent residents of the selected households were interviewed to collect information on sociodemographic characteristics, household WASH conditions (based on WHO/UNICEF JMP indicators), and hygiene‐related practices, including facial cleanliness, frequency of face washing, and soap use.

### 2.4. Inclusion and Exclusion Criteria

Households were eligible if they had lived in the selected kebeles for at least 6 months prior to data collection and provided informed consent. Households without a child aged 1–9 years were excluded. Children who were absent after two revisits were excluded from clinical examination. Caregivers who were seriously ill or unable to communicate during data collection were also excluded.

### 2.5. Sample Size Determination

The sample size was calculated using the single population proportion formula with the following assumptions: a 95% confidence level, 5% margin of error, and an expected prevalence of 23.4%, based on a previous study conducted in Ethiopia [17].

Based on the calculation *n* = *Z* ^2^ *p*(1 − *p*)/*d* ^2^ [18], the initial sample size was 276. A design effect of 1.5 was applied to account for the cluster sampling design. The sample size was then adjusted to account for a 10% nonresponse rate, resulting in a final sample size of 456 households (456 children aged 1–9 years).

### 2.6. Sampling Procedure

A multistage sampling technique was employed to select study participants. Initially, North Gondar Zone, which comprises seven woredas, was used as the primary sampling frame. From these, four woredas were selected using a simple random sampling method to ensure equal probability of inclusion.

Within the selected woredas, a total of 16 rural kebeles (four kebeles from each woreda) were selected using probability proportional to size (PPS) sampling to ensure representativeness of the study population and to account for variations in population size across kebeles.

A household sampling frame was obtained from health extension workers in each selected kebele. The total sample size was then proportionally allocated to each kebele based on the number of eligible households. Households were selected using systematic random sampling. The sampling interval (*k*) was calculated by dividing the total number of eligible households in each kebele by the allocated sample size. The first household was selected using a simple random start, and thereafter every kth household was included until the required sample size was achieved.

Within each selected household, one child aged 1–9 years was included for clinical examination. In households with more than one eligible child, one child was selected using simple random sampling to ensure equal probability of inclusion and to avoid potential intrahousehold clustering bias.

This multistage sampling approach introduced clustering at the woreda, kebele, and household levels; therefore, appropriate adjustments (a design effect) were applied during sample size determination to account for potential intracluster correlation.

### 2.7. Study Variables

The dependent variable of the study was active trachoma, defined as the presence of trachomatous inflammation–follicular (TF) and/or trachomatous inflammation–intense (TI) in either eye according to the WHO simplified trachoma grading system. TF and TI are graded independently and may co‐occur in the same child, either in different eyes or within the same eye according to the WHO simplified grading system.

The independent variables included sociodemographic characteristics, environmental factors, WASH indicators, and hygiene‐related practices. Sociodemographic variables comprised age, sex, caregiver educational status, occupation, household size, and household socioeconomic status. Household socioeconomic status was assessed using a wealth index derived from principal component analysis (PCA) of selected household assets and housing characteristics and categorized into low‐, medium‐, and high‐wealth tertiles.

WASH‐related variables included the type of household water source, travel time to collect water, water use for hygiene, latrine availability, reported latrine utilization, household waste disposal practices, and household crowding. Water sources were classified as improved or unimproved according to WHO/UNICEF JMP definitions. Travel time to collect water was categorized as < 30 min or ≥ 30 min round trip in accordance with JMP recommendations. Household crowding was assessed using the person‐to‐room ratio and categorized based on predefined cutoff values.

Water use for hygiene was assessed based on caregivers’ perceived sufficiency of household water for routine daily hygiene activities and categorized as adequate or inadequate. This variable reflects caregivers’ subjective perception rather than objectively measured water quantity. This measure was not based on a validated or standardized measurement scale but was obtained through a single caregiver perception‐based question. Water availability refers to the frequency and regularity of household water supply (daily vs. irregular), whereas water use for hygiene reflects caregivers’ perceived sufficiency of available water for routine daily hygiene activities.

Environmental variables included fly density around the child’s face. Fly density was defined as high when ≥ 3 eye‐seeking flies were observed on or around the child’s face within approximately 30 s during household assessment; otherwise it was classified as low (< 3 flies).

Hygiene‐related variables included caregiver‐reported face‐washing practices and caregiver‐reported use of soap during the child’s face washing, as well as observed household soap availability. These variables were considered indicators or proxies of facial cleanliness rather than direct measures of behavioral determinants.

Face‐washing practice was assessed as a composite indicator of facial cleanliness. Good practice was defined as washing the child’s face ≥ 2 times per day and the absence of ocular or nasal discharge, while poor practice was defined as washing < 2 times per day or the presence of ocular or nasal discharge.

### 2.8. Data Collection

Data were collected using a structured questionnaire adapted from the WHO trachoma assessment guidelines, the WHO/UNICEF JMP indicators for WASH, and relevant published literature [19, 20]. The JMP framework served as the standard reference for classifying water source and sanitation indicators to facilitate comparability with international monitoring standards. The questionnaire was administered to caregivers and captured information on sociodemographic characteristics, household environmental conditions, WASH access, and hygiene‐related practices.

Information on the primary household water source was collected and categorized according to WHO/UNICEF JMP definitions. Improved water sources included piped water, public taps or standpipes, boreholes, protected springs, and protected dug wells. Unimproved water sources included unprotected springs, unprotected dug wells, rivers, streams, open ponds, and other surface water sources. Travel time to collect water was recorded and categorized as < 30 or ≥ 30 min round trip. Information on water availability for household use was also collected to reflect conditions influencing hygiene practices.

Additional data were collected on latrine availability, caregiver‐reported latrine utilization, household waste disposal practices, household crowding, soap availability, face‐washing practices, and soap use during children’s face washing. Soap availability referred to the observed presence of soap in the household at the time of the survey. Household waste disposal practices were categorized as proper waste disposal, defined as disposal into covered pits, compost pits, designated household waste pits, or organized collection systems, and open field disposal, defined as dumping or burning household waste in open spaces, roadsides, riverbanks, or other uncontrolled environments without sanitary containment.

Fly density was assessed through direct observation of eye‐seeking flies on or around the child’s face during household visits. Fly density was categorized as high when ≥ 3 eye‐seeking flies were observed on or around the child’s face within 30 s during observation; otherwise it was classified as low.

Clinical eye examinations were conducted among children aged 1–9 years under adequate natural light using the WHO simplified trachoma grading system. Prior to field deployment, eye care professionals received standardized training based on WHO protocols, including both theoretical and practical components focused on the identification of TF and TI. Particular emphasis was placed on differentiating TF from other causes of follicular or nontrachomatous conjunctivitis, including viral, bacterial, and allergic conjunctivitis. Training incorporated WHO standardized photographic grading charts, case‐based demonstrations, and supervised practical exercises.

Competency assessments were conducted before data collection to ensure diagnostic accuracy and consistency. Interobserver agreement among examiners was evaluated prior to fieldwork, and good agreement was achieved (*κ* = 0.82). Standard infection prevention procedures were strictly followed throughout the data collection process.

### 2.9. Data Quality Assurance

Data collectors and supervisors received intensive training on the study protocol, interview techniques, and trachoma grading procedures. The questionnaire was pretested in a nonselected kebele, and necessary modifications were made.

Daily supervision and review of completed questionnaires ensured completeness and consistency. WHO diagnostic standards were strictly followed to minimize measurement error.

### 2.10. Data Analysis

Data were entered into EpiData Version 4.6 and exported to SPSS Version 30 for statistical analysis. Descriptive statistics, including frequencies, percentages, means, and standard deviations, were used to summarize sociodemographic, environmental, WASH, and hygiene‐related characteristics of the study participants.

Household socioeconomic status was constructed using PCA based on selected household asset ownership and housing characteristics. The resulting wealth index scores were categorized into low, medium, and high tertiles and used as a proxy indicator of socioeconomic status in all analyses. Household crowding was defined as the presence of more than three persons per sleeping room and categorized as high (> 3 persons/room) or low (≤ 3 persons/room). Fly density was assessed through direct observation during household visits and categorized as high (≥ 3 eye‐seeking flies observed on or around the child’s face within approximately 30 s) or low (< 3 flies), consistent with previous trachoma studies.

WASH variables were defined according to the WHO/UNICEF JMP classification framework (2021). Accordingly, improved water sources included piped water, boreholes, protected wells, protected springs, and public standpipes, whereas unimproved sources included unprotected wells, unprotected springs, and surface water sources. Key water access indicators, including household water availability and travel time to water collection points, were used as proxy measures of water access constraints.

Bivariable logistic regression analyses were performed to assess associations between independent variables and active trachoma. Variables with *p* value < 0.25, along with biologically plausible covariates (child age, sex, and household socioeconomic status), were entered into the multivariable logistic regression model. Water access and sanitation variables were also included a priori due to their established relevance to trachoma transmission.

In the multivariable logistic regression model, adjusted odds ratios (AORs) with 95% confidence intervals (CIs) were reported, and statistical significance was set at *p* < 0.05. Multicollinearity was assessed using the variance inflation factor (VIF < 2.5), and model fit was evaluated using the Hosmer–Lemeshow goodness‐of‐fit test. Potential interaction effects between water access variables and sociodemographic factors (age, sex, and socioeconomic status) were tested, but none were statistically significant and, therefore, were not retained.

Although a multistage cluster sampling design was used, clustering was accounted for in sample size estimation through a design effect. However, regression analysis was performed using a single‐level logistic model without multilevel or cluster‐robust standard errors. Given the relatively small number of clusters and the focus on individual‐level associations, observations were treated as independent in the final analysis.

## 3. Results

### 3.1. Sociodemographic Characteristics of Study Participants

The sociodemographic characteristics of study participants are summarized in Table 1. A total of 456 caregivers of children aged 1–9 years were included in the study. The age distribution of caregivers showed that nearly half (48.5%) were aged 30–44 years, followed by 28.9% aged 18–29 years and 22.6% aged 45 years and above. The majority of respondents were female (63.8%), while males accounted for 36.2%.

**TABLE 1:** Sociodemographic characteristics of study participants in rural communities of North Gondar Zone, Ethiopia (*n* = 456).

Variable	Category	Frequency (*n*)	Percentage (%)
Age of caregiver	18–29	132	28.9
	30–44	221	48.5
	≥ 45	103	22.6

Sex	Male	165	36.2
	Female	291	63.8

Educational status	No formal education	247	54.2
	Primary	134	29.4
	Secondary and above	75	16.4

Occupation	Farmer	287	62.9
	Housewife	104	22.8
	Other	65	14.3

Household size	≤ 4	168	36.8
	5–7	231	50.7
	≥ 8	57	12.5

Wealth status	Low	241	52.9
	Medium	145	31.8
	High	70	15.3

Regarding educational status, more than half (54.2%) had no formal education, while 29.4% had completed primary education and 16.4% had secondary education or higher. In terms of occupation, most caregivers were farmers (62.9%), followed by housewives (22.8%) and others (14.3%).

Household size was predominantly five to seven members (50.7%), followed by four or fewer members (36.8%) and eight or more members (12.5%). More than half of the households (52.9%) were classified as having low socioeconomic status, while 31.8% and 15.3% were medium and high, respectively.

### 3.2. Household WASH and Environmental Characteristics

The household WASH, and environmental characteristics of the study participants are presented in Table 2. Regarding water access, 274 (60.1%) households relied on unimproved water sources, while 182 (39.9%) had access to improved water sources. In accordance with WHO/UNICEF JMP classifications, unimproved water sources included unprotected springs, unprotected dug wells, open ponds, rivers, streams, and other surface water sources, whereas improved sources included protected springs, boreholes, piped water supplies, and public taps or standpipes.

**TABLE 2:** Household water, sanitation, hygiene, and environmental characteristics in rural communities of North Gondar Zone, Ethiopia (*n* = 456).

Variable	Category	Frequency (*n*)	Percentage (%)
Water source	Improved	182	39.9
	Unimproved	274	60.1

Water availability	Daily	154	33.8
	Irregular	302	66.2

Travel time to collect water (round trip)	< 30 min	198	43.4
	≥ 30 min	258	56.6

Latrine availability	Yes	280	61.4
	No	176	38.6

Reported latrine utilization	Yes	258	56.6
	No	198	43.4

Handwashing facility availability	Yes	140	30.7
	No	316	69.3

Observed soap availability	Yes	169	37.1
	No	287	62.9

Waste disposal method	Proper	144	31.6
	Open field	312	68.4

Fly density	Low	177	38.8
	High	279	61.2

Household crowding	Low	261	57.2
	High	195	42.8

*Note:* High fly density was defined as the observation of ≥ 3 eye‐seeking flies on or around the child’s face within approximately 30 s during household assessment; low fly density was defined as < 3 flies. Household crowding was classified using the person‐to‐room ratio, with high crowding defined as more than three persons per sleeping room and low crowding as three or fewer persons per sleeping room. Soap availability was defined as the presence of soap observed during household visits.

A total of 302 (66.2%) households reported irregular water availability. More than half of the respondents, 258 (56.6%), reported spending 30 min or more on a round trip to collect water, indicating limited accessibility that may constrain household hygiene practices.

Regarding sanitation, 280 (61.4%) households reported having latrine facilities, while 176 (38.6%) had no latrine access. Among all respondents, 198 (43.4%) reported that household latrines were not regularly utilized. Most households lacked basic hygiene infrastructure, with 316 (69.3%) having no handwashing facilities and 287 (62.9%) having no soap observed during household visits.

Environmental sanitation conditions were generally poor. Open‐field waste disposal was reported by 312 (68.4%) households, whereas 144 (31.6%) practiced proper waste disposal. High fly density was observed in 279 (61.2%) households, while 177 (38.8%) had low fly density. In addition, 195 (42.8%) households experienced high household crowding based on the person‐to‐room ratio.

### 3.3. Hygiene‐Related Practices and Environmental Conditions

The hygiene‐related practices and environmental conditions of the study households are presented in Table 3. Overall, 261 (57.2%) caregivers reported poor face‐washing practices among children, while 195 (42.8%) reported good face‐washing practices. Good face‐washing practice was defined as washing the child’s face at least twice per day and the absence of ocular or nasal discharge, whereas poor practice was defined as washing less than twice per day or the presence of ocular or nasal discharge. Caregiver‐reported soap use during child face washing was low, with only 107 (23.5%) households reporting soap use, whereas 349 (76.5%) reported that soap was not used.

**TABLE 3:** Hygiene‐related practices and environmental conditions among households in rural communities of North Gondar Zone, Ethiopia (*n* = 456).

Variable	Category	Frequency (*n*)	Percentage (%)
Face‐washing practice	Good	195	42.8
	Poor	261	57.2

Caregiver‐reported soap use during child face washing	Yes	107	23.5
	No	349	76.5

Water use for hygiene	Adequate	195	42.8
	Inadequate	261	57.2

Ocular/nasal discharge in children	Yes	208	45.6
	No	248	54.4

Fly density	Low	177	38.8
	High	279	61.2

*Note:* Fly density was defined as high when ≥ 3 eye‐seeking flies were observed on or around the child’s face within approximately 30 s during household assessment; otherwise, it was classified as low. Water use for hygiene reflects caregivers’ perceived sufficiency of household water for daily hygiene activities and was categorized as adequate or inadequate.

More than half of the households, 261 (57.2%), reported inadequate water use for hygiene purposes, while 195 (42.8%) reported adequate use. Ocular or nasal discharge was observed or reported among 208 (45.6%) children. High fly density was observed in 279 (61.2%) households, while low fly density was observed in 177 (38.8%) households (Table 3).

### 3.4. Prevalence of Active Trachoma

A total of 456 children aged 1–9 years were examined for clinical signs of trachoma using the WHO simplified trachoma grading system. Active trachoma was defined as the presence of TF and/or TI in at least one eye.

Overall, 94 children were diagnosed with active trachoma, giving a prevalence of 20.6% (95% CI: 16.9%–24.3%). Among the examined children, 69 (15.1%) had TF, and 25 (5.5%) had TI. TF and TI are distinct clinical signs that may coexist in the same child, and classification follows the WHO simplified trachoma grading system.

**FIGURE 1:**
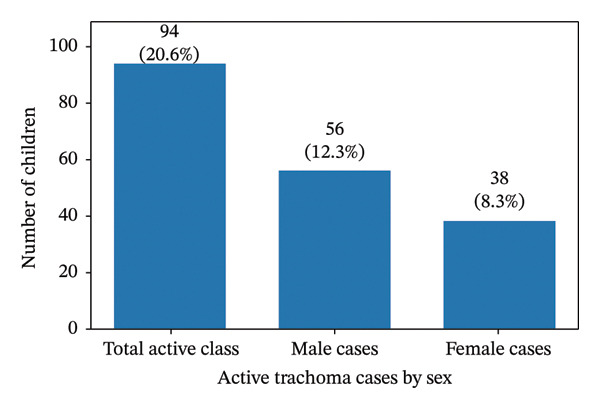
Prevalence of active trachoma by sex among children aged 1–9 years in rural communities of North Gondar Zone, Ethiopia (*n* = 456).

Regarding sex distribution, 56 of the 94 cases (59.6%) were males, and 38 (40.4%) were females. Among all examined children, 12.3% were male cases and 8.3% were female cases, indicating a higher burden of active trachoma among male children (see Table 4) (see Figure 1).

**TABLE 4:** Prevalence of active trachoma among children aged 1–9 years (*n* = 456).

Indicator	Value
Total examined children	456
TF cases	69 (15.1%)
TI cases	25 (5.5%)
Total active trachoma cases	94 (20.6%)
95% CI for prevalence	16.9%–24.3%

*Note:* TF, trachomatous inflammation–follicular; TI, trachomatous inflammation–intense.

### 3.5. Age‐Specific Distribution of Active Trachoma

The age‐specific distribution of active trachoma among children aged 1–9 years is presented in Table 5. A total of 94 cases were distributed unevenly across age groups, with a higher burden observed in younger children.

**TABLE 5:** Age‐specific distribution of TF and TI cases among children aged 1–9 years (*n* = 456).

Age (years)	Examined (*n*)	TF *n*	TI *n*	Total active trachoma *n* (%)
1	42	10	4	14 (33.3)
2	50	11	4	15 (30.0)
3	55	10	3	13 (23.6)
4	52	9	3	12 (23.1)
5	51	8	2	10 (19.6)
6	49	7	2	9 (18.4)
7	53	6	2	8 (15.1)
8	52	5	1	6 (11.5)
9	52	3	4	7 (13.5)
Total	456	69	25	94 (20.6)

*Note:* TF, trachomatous inflammation–follicular. TI, trachomatous inflammation–intense.

The highest prevalence was recorded at age 1 year (33.3%) and age 2 years (30.0%). A gradual decline in prevalence was observed with increasing age, reaching the lowest level at age 8 years (11.5%), followed by a slight increase at age 9 years (13.5%). TF cases were consistently more frequent than TI across all age groups. Overall, the findings indicate that younger children carry a higher burden of active trachoma compared to older age groups.

### 3.6. Bivariable and Multivariable Logistic Regression Analysis

Bivariable logistic regression analysis was conducted to assess the association between independent variables and active trachoma. Variables with a *p* value < 0.25, along with biologically plausible covariates (child age, sex, and household wealth index), were entered into the multivariable logistic regression model to control for confounding. Crude and adjusted odds ratios (CORs and AORs), with 95% CIs, were reported.

In the bivariable analysis, unimproved water source, lack of latrine availability, poor face‐washing practice, caregiver‐reported absence of soap use, and high fly density were associated with active trachoma. After adjustment, unimproved water source, absence of latrine, poor face‐washing practice, and high fly density remained independent predictors, while caregiver‐reported soap use did not retain statistical significance in the multivariable model.

Children from households using unimproved water sources had higher odds of active trachoma (*A* *O* *R* = 1.76; 95% CI: 1.01–3.05). Lack of latrine availability was also associated with increased odds of infection (*A* *O* *R* = 1.94; 95% CI: 1.15–3.28). Poor face‐washing practice showed a strong association with active trachoma (*A* *O* *R* = 2.63; 95% CI: 1.52–4.55), while high fly density remained a significant predictor (*A* *O* *R* = 2.71; 95% CI: 1.60–4.58).

Caregiver‐reported soap use was not significantly associated with active trachoma after adjustment (*A* *O* *R* = 1.18; 95% CI: 0.36–2.01; *p* = 0.076). The wide CI indicates limited precision of the estimate, and this result should therefore be interpreted with caution (Table 6).

**TABLE 6:** Bivariable and multivariable logistic regression analysis of factors associated with active trachoma among children aged 1–9 years in rural communities of North Gondar Zone, Ethiopia (*n* = 456).

Variable	Category	Trachoma + *n* (%)	Trachoma − *n* (%)	COR (95% CI)	*p* value	AOR (95% CI)	*p* value
Water source	Improved	24 (5.3)	158 (34.6)	1.00 (Ref)	—	1.00 (Ref)	—
	Unimproved	70 (15.4)	204 (44.7)	2.26 (1.36–3.76)	< 0.001	1.76 (1.01–3.05)	0.045

Latrine availability	Yes	38 (8.3)	242 (53.1)	1.00 (Ref)	—	1.00 (Ref)	—
	No	56 (12.3)	120 (26.3)	2.97 (1.86–4.74)	< 0.001	1.94 (1.15–3.28)	0.013

Face‐washing practice	Good	26 (5.7)	182 (39.9)	1.00 (Ref)	—	1.00 (Ref)	—
	Poor	68 (14.9)	180 (39.5)	2.65 (1.61–4.35)	< 0.001	2.63 (1.52–4.55)	< 0.001

Soap use	Yes	14 (3.1)	93 (20.4)	1.00 (Ref)	—	1.00 (Ref)	—
	No	80 (17.5)	269 (59.0)	1.98 (1.07–3.67)	0.029	1.18 (0.36–2.01)	0.076

Fly density	Low	20 (4.4)	165 (36.2)	1.00 (Ref)	—	1.00 (Ref)	—
	High	74 (16.2)	197 (43.2)	3.10 (1.81–5.29)	< 0.001	2.71 (1.60–4.58)	< 0.001

*Note:* Crude odds ratios (CORs) were obtained from bivariable logistic regression. Variables with *p* < 0.25 and biologically plausible covariates were included in the multivariable model. Adjusted odds ratios (AORs) were controlled for child age, sex, household wealth index (PCA‐derived), water source type, household water availability, travel time to water source, latrine availability, fly density, and hygiene‐related practices. Model diagnostics indicated no multicollinearity among variables (VIF < 2.5), and the Hosmer–Lemeshow test confirmed good model fit.

## 4. Discussion

This community‐based study assessed the prevalence of active trachoma and its associated WASH and hygiene‐related factors among children aged 1–9 years in rural North Gondar Zone, Ethiopia. The overall prevalence of active trachoma was 20.6%, which is substantially higher than the WHO elimination threshold of 5% [21], indicating ongoing transmission and confirming that trachoma remains a major public health concern in the study area.

The prevalence observed in this study is consistent with reports from other highly endemic regions of Ethiopia, particularly Amhara and Oromia, where similarly high levels of infection have been documented [22–24]. Comparable findings have also been reported in several sub‐Saharan African countries, including Niger, Mali, and South Sudan, where trachoma persists in rural and resource‐limited settings [25–27]. However, the prevalence is higher than that reported from districts where sustained implementation of the SAFE strategy has reduced trachoma levels to 10%–15% [28, 29]. These differences may reflect variations in the coverage and consistency of SAFE interventions, particularly sanitation improvement, facial cleanliness promotion, and environmental control measures [30].

Recent evidence supports these findings. A community‐based Ethiopian study reported persistently high trachoma prevalence despite SAFE implementation, with strong associations between poor sanitation and hygiene conditions [24]. Similarly, another Ethiopian study highlighted the continued importance of latrine absence, fly density, and environmental contamination in sustaining transmission [31]. A systematic review and meta‐analysis further confirmed that improved sanitation, facial cleanliness, and access to water are consistently protective against active trachoma [19]. Collectively, these findings indicate that North Gondar Zone remains a highly endemic setting requiring strengthened control efforts.

A higher prevalence of active trachoma was observed among male children compared to females. This difference may reflect differential exposure to environmental risks such as outdoor play and animal contact among boys. However, this interpretation should be cautious because sex‐specific age distributions were not separately assessed, and residual confounding by age may partly explain this difference. In addition, no formal interaction analysis between age and sex was conducted.

Children from households using unimproved water sources had higher odds of active trachoma. Although water source type remained significant in the multivariable model, other water access indicators such as travel time and reported household water availability did not independently explain the association. This suggests that unimproved water sources may reflect broader structural inequalities in WASH conditions rather than water access alone [19]. Residual confounding from unmeasured household and behavioral factors may also contribute to this association.

Lack of latrine facilities was significantly associated with higher odds of active trachoma. This is likely to contribute to environmental contamination and may create conditions favorable for eye‐seeking fly proliferation. In rural settings, the absence of latrines is commonly associated with open defecation, which increases transmission risk [32].

High fly density was independently associated with active trachoma, likely reflecting poor environmental sanitation, including unmanaged waste and fecal contamination that provide breeding sites for flies [31]. Increased fly–human contact, particularly with children’s faces, may facilitate repeated transmission of ocular infection in settings with limited hygiene infrastructure [33, 34].

Poor face‐washing practice was strongly associated with active trachoma. Children with unclean faces, particularly those with ocular or nasal discharge, are more likely to attract flies and experience repeated exposure to infection [35]. This underscores the importance of consistent facial cleanliness as a key preventive strategy and aligns with evidence emphasizing facial cleanliness as a core component of the SAFE strategy.

Although caregiver‐reported soap use showed a crude association in bivariable analysis, it was not statistically significant after adjustment in the multivariable model. This may reflect reporting bias and social desirability effects. It also suggests that soap use alone may not be sufficient without consistent water availability and regular facial cleanliness practices.

Age, sex, and household socioeconomic status were included in the multivariable model to control for confounding based on established epidemiological evidence. However, residual confounding may still exist due to limitations in measuring socioeconomic gradients and behavioral heterogeneity, which may influence both exposure and hygiene practices.

The burden of active trachoma may also be spatially heterogeneous, as previous studies have demonstrated clustering at household and village levels [36]. The absence of geospatial data limited the identification of transmission hotspots. Future studies incorporating spatial analysis could improve targeting of SAFE interventions.

Overall, the findings suggest that active trachoma in North Gondar Zone is driven by interacting environmental and behavioral factors rather than a single determinant. Despite ongoing SAFE strategy implementation, important gaps remain in sustained sanitation coverage, consistent facial cleanliness, and broader structural WASH conditions.

The persistently high burden may also reflect contextual factors such as limited access to health services in remote areas, seasonal variation in water availability, and entrenched hygiene practices. Since data were collected during the dry season (January–April 2025), the findings likely reflect seasonal constraints affecting water availability and fly density rather than year‐round conditions. Therefore, longitudinal studies covering both dry and wet seasons are recommended to better capture temporal variation in trachoma risk factors.

### 4.1. Limitations of the Study

This study has several limitations that should be considered when interpreting its findings. First, the cross‐sectional design limits the ability to establish causal relationships between the identified risk factors and active trachoma, as exposure and outcome were assessed simultaneously.

In addition, much of the behavioral data relied on caregiver self‐reports, which may be subject to recall bias and social desirability bias, particularly for hygiene‐related practices such as facial cleanliness, handwashing, and soap use. As a result, some behaviors may have been either overreported or underreported. Although standardized WHO trachoma grading criteria were used and good interobserver agreement was achieved (kappa = 0.82), a small degree of observer bias cannot be excluded.

The study also did not account for seasonal variations in key environmental and behavioral factors, including water availability, sanitation practices, and fly density, all of which are known to influence trachoma transmission dynamics. Data collection was conducted exclusively during the dry season (January–April 2025), which may limit the generalizability of the findings across different seasons. Consequently, the observed associations may either underestimate the effects of water scarcity experienced in other periods of the year or overestimate the influence of dry‐season fly density.

Furthermore, the study did not include spatial or geospatial analysis due to the absence of household geographic coordinates. Although a multistage sampling approach was used to enhance representativeness, the lack of spatial data prevented the identification of potential clustering patterns or transmission hotspots at the village or household levels. Given the known spatial heterogeneity of trachoma transmission, future studies incorporating geospatial methods are recommended to support more targeted intervention strategies.

Another important limitation is that direct measurements of household water quantity for hygiene purposes were not collected. Although information was gathered on water source type, availability, and travel time, the actual volume of water used for hygiene could not be quantified. In addition, water use for hygiene was based on caregivers’ perceived sufficiency of household water for daily hygiene activities rather than on an objective measurement, making it a subjective indicator that may introduce reporting bias.

Finally, although children diagnosed with active trachoma were referred for treatment, there was no mechanism to follow up on whether treatment was actually received or completed. This limits the ability to assess treatment uptake and adherence.

Overall, these limitations suggest that the findings should be interpreted with caution. Future studies incorporating longitudinal designs, seasonal comparisons, geospatial mapping, and follow‐up of treatment outcomes would provide a more comprehensive understanding of trachoma transmission dynamics in the area.

## 5. Conclusion and Recommendations

Active trachoma remains a significant public health problem in rural North Gondar Zone, with a prevalence of 20.6% among children aged 1–9 years, which is far above the WHO elimination threshold. The study identified key modifiable risk factors, including unimproved water sources, lack of latrine facilities, poor facial hygiene practices, and high fly density, all of which were associated with increased odds of active trachoma.

Overall, the findings indicate that both environmental and behavioral factors continue to play an important role in sustaining trachoma transmission in the area. These results also highlight persistent gaps in the full and effective implementation of the WHO SAFE strategy at the community level.

Integrated and sustained implementation of the WHO SAFE strategy is essential in rural North Gondar Zone. Priority should be given to improving access to safe and reliable water sources, expanding functional latrine coverage, and strengthening community‐based promotion of facial cleanliness among children. In addition, environmental sanitation measures and fly control interventions should be reinforced to reduce exposure risk and support long‐term trachoma elimination efforts.

## Author Contributions

G.A.A. was responsible for the overall study, including conceptualization, study design, data collection, data analysis, interpretation of results, and manuscript writing.

## Funding

This study did not receive any external funding.

## Disclosure

The author reviewed and approved the final version of the manuscript.

## Ethics Statement

Ethical approval for this study was obtained from the Debark University Research Ethics Committee (Ref. No: REC/DKU/02/2025). Permission was also secured from the North Gondar Zone Health Department and local administrative offices. The study was conducted in accordance with the Declaration of Helsinki.

Written informed consent was obtained from caregivers of all participating children after explaining the study objectives, procedures, risks, benefits, and voluntary nature of participation. For older children, verbal assent was obtained in addition to caregiver consent where appropriate.

Children diagnosed with active trachoma were referred to nearby health facilities and health extension workers for further evaluation and management according to national SAFE strategy guidelines. However, no formal follow‐up was conducted to verify treatment uptake, as this was beyond the scope of the study.

All data were kept confidential, used only for research purposes, and analyzed anonymously without personal identifiers.

## Consent

Please see Ethics Statement.

## Conflicts of Interest

The author declares no conflicts of interest.

## Data Availability

The datasets generated and/or analyzed during this study are available from the corresponding author upon reasonable request.
